# 2022 ISCB Overton Prize: Po-Ru Loh

**DOI:** 10.1093/bioinformatics/btac338

**Published:** 2022-06-27

**Authors:** Christina Fogg, Diane Kovats, Martin Vingron

**Affiliations:** Freelance Writer, Kensington, MD, USA; International Society for Computational Biology, Leesburg, VA, USA; Max Planck Institute for Molecular Genetics, Berlin, Germany

The ISCB Overton recognizes the research, education, and service accomplishments of early or mid-career scientists who are emerging leaders in computational biology and bioinformatics. The Overton Prize was established in 2001 to honor the untimely loss of G. Christian Overton, a leading bioinformatics researcher and a founding member of the ISCB Board of Directors. The 2022 Overton Prize winner is Dr. Po-Ru Loh, Assistant Professor in the Division of Genetics and Center for Data Sciences, Brigham and Women’s Hospital and Harvard Medical School, and Associate Member of the Broad Institute of MIT and Harvard. He will receive his award and give a keynote address at the 30^th^ Conference on Intelligent Systems for Molecular Biology (ISMB) in Madison, WI being held from July 10-14, 2022.

*Po-Ru Loh: From Math Olympiad to Millions of Genomes*


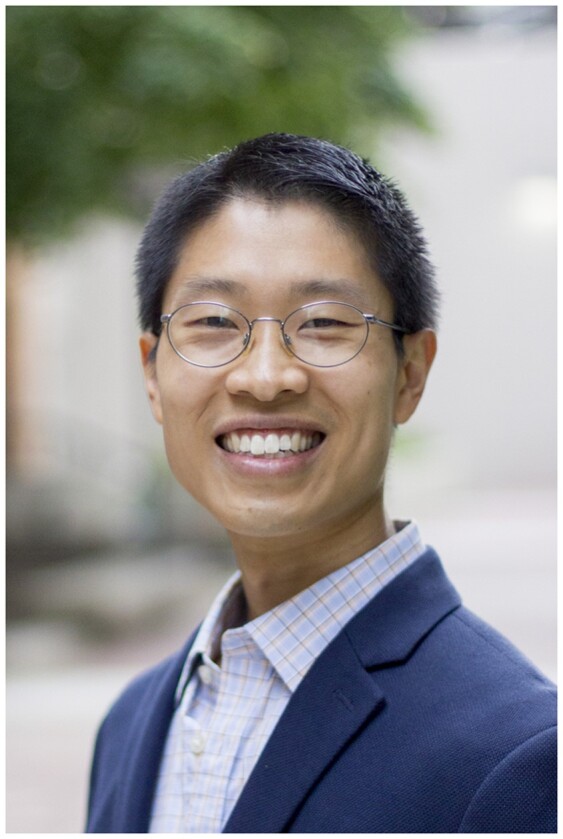


Po-Ru Loh grew up in Madison, WI and was encouraged to study arithmetic and algebra from a young age. He recalls liking mathematics in school because of his familiarity with the subject matter, and he also developed an interest in solving mathematical puzzles and competing with his older brother to solve riddles in Brian Bolt’s *Mathematical Funfair* book. As a middle and high school student, Loh’s interest in math was further stoked by math competitions, including MathCounts and the Math Olympiad, and he went on to be a 2002 and 2003 Gold medalist, and top scorer on US team in the International Mathematical Olympiad. However, Loh’s early encounters with science were somewhat discouraging, as he recalled: “My first experiences with science involved growing lima beans and mealworms. My beans didn’t sprout, and my mealworms died. It wasn’t until high school that I realized that science had a quantitative side, and I became more interested.”

Loh received a B.S. in mathematics from the California Institute of Technology in 2007, during which time he was a second-place finisher in both the Google Code Jam and the TopCoder Open. He strongly considered being a pure mathematician as an undergraduate but eventually realized he wanted to work on problems with more direct real-world relevance. Loh ended up pursuing a Ph.D. in applied mathematics at MIT and considers his entry into computational biology to be somewhat accidental. He said, “I had no idea what field of study to pursue, so upon matriculating, I simply browsed through faculty research interests and spent my first year sampling an eclectic mix of courses in different fields that sounded interesting. Computational biology was one of them, and it caught my eye as a growing field with interesting algorithmic challenges.” Loh joined Bonnie Berger’s lab and went on to develop a dissertation project focused on compressive genomics. He worked on algorithms that computed directly on compressed genomic data, which allowed for analyses to keep pace with data generation.

In 2013, Loh began his postdoc under the mentorship of Alkes Price at Harvard T.H. Chan School of Public Health and dove further into computational genetics. With Price, he pioneered ultra-efficient algorithms facilitating biobank-scale genomics through the development of two widely used computational genetics tools, BOLT-LMM and Eagle2. These tools have been used to analyze millions of genomes and have brought to light numerous loci that shape human health and disease. Loh became an Assistant Professor in the Division of Genetics and Center for Data Sciences, Brigham and Women’s Hospital and Harvard Medical School, and Associate Member of the Broad Institute of MIT and Harvard in 2018. During his time as a postdoc and a tenure-track faculty member, Loh has had some unexpected findings that have impacted his research. He said, “The most surprising findings in my research thus far have been unexpectedly strong associations between inherited genetic variants and various human traits, ranging from height to clonal hematopoiesis. Prior to these projects, I had always thought of myself as a tool-builder. I developed statistical methods to help answer questions in genetics, but I left the application of these methods to the “real” geneticists. I was quite shocked the first time that my analyses uncovered new biological knowledge that neither I nor any of my collaborators expected. But these new findings made sense, could be validated, and were ultimately very satisfying. These experiences have shifted my path substantially, increasing my appetite for taking on projects driven by biological questions rather than only method development, and leading me to pursue several projects investigating genomic structural variation.”

Loh’s current interests include studying very rare coding variants and genomic structural variants using computational methods that leverage haplotype sharing withing biobank cohorts, as well as developing methods to detect mosaic chromosomal alterations and understand how they relate to cancer and other genetic disorders. He is irrepressibly enthusiastic and curious and said, “At the moment, I am particularly intrigued by the potential to leverage population-scale whole-genome sequencing to learn more about genomic variants that have typically been difficult to ascertain – specifically, structural variants and somatic variants. However, I would not be surprised if I find myself working on entirely new research directions five years from now. My trajectory thus far has been greatly influenced by serendipitous encounters, and given the rate at which new “omic” data sets and data types become available, it seems essential to keep an eye out for important new resources and the challenges and opportunities they bring.”

Loh is deeply appreciative of his mentors Berger and Price, and he recognizes their investment in him as a scientist, which included helping him identify projects that leveraged his existing skills, pushing him to expand his skills and knowledge, and helping him chart a path to independence. Soumya Raychaudhuri and Richard Maas have also been instrumental in guiding Loh through the establishment of his independent research group. His mentorship has deeply shaped how he works with graduate students and postdocs in his lab.

As an early career scientist, Loh has been extremely productive, with over 70 publications, >17,000 citations and numerous awards and fellowships. Loh has consistently developed open-source software tools that are used by the computational biology community, and he has served on program committees for ISMB and RECOMB. Loh is particularly grateful for his recognition with the Overton Prize and said, “It is an incredible honor to receive this award. When I first attended the ISMB conference as a graduate student ten years ago, I never imagined I could one day be selected for the Overton Prize. I am tremendously grateful to all the mentors, collaborators, and trainees who contributed to the work recognized by this award and to my development as a scientist.”

